# Molecular basis and therapeutic potential of sortase A inhibitors as new antibiotics against MDR pathogens

**DOI:** 10.1098/rsob.250415

**Published:** 2026-09-09

**Authors:** Ananya Dash, Christopher LaRock

**Affiliations:** ^1^ Immunology and Molecular Pathogenesis Graduate Program, Emory University, Atlanta, GA, USA; ^2^ Department of Microbiology and Immunology, Emory University School of Medicine, Atlanta, GA, USA; ^3^ Division of Infectious Diseases, Emory University School of Medicine, Atlanta, GA, USA

**Keywords:** antibiotic discovery, anti-infective, sortase, infectious disease, Gram-positive

## Abstract

Attaching proteins to the cell surface presents a technical challenge to Gram-positive bacteria. A class of enzymes called sortases solves this challenge by covalently anchoring proteins, including an arsenal of virulence factors, to the cell wall. Most Gram-positive bacteria have multiple critical factors that are anchored in this manner and, coupled with the extracellular location of this reaction, make sortase an attractive target for the development of antibiotics. However, since sortase is neither essential nor itself a virulence factor, it is a very different sort of target compared to those investigated in other antibiotic strategies. This review explores the challenges of treating infection by Gram-positive pathogens, the importance of sortase-anchored proteins in the host–pathogen interaction and the known and probable antimicrobial effects of sortase inhibition for infections by *Staphylococcus aureus, Streptococcus pyogenes, Bacillus anthracis, Listeria monocytogenes* and other Gram-positive pathogens.

## 1. Introduction

**Figure 1.**
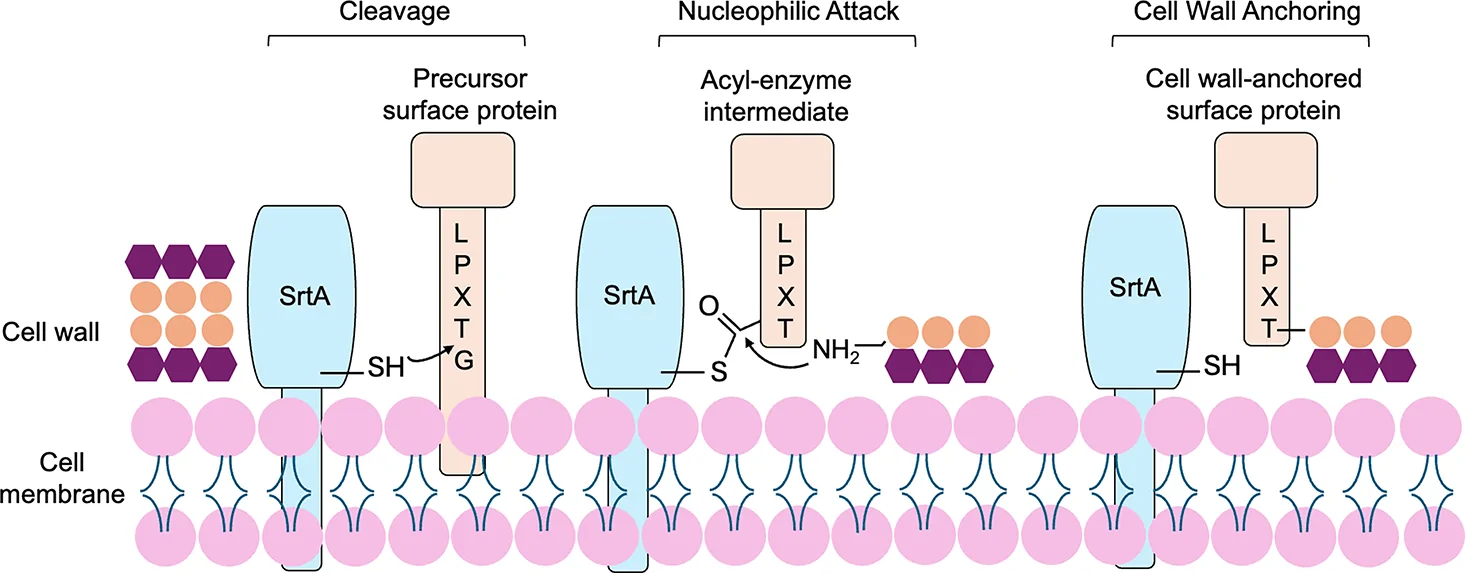
Sortase A anchors surface proteins on the cell wall of Gram-positive bacteria. Cleavage: Sortase A recognizes precursor surface protein containing a carboxy-terminal amino acid motif LPXTG, where ‘X’ tolerates most substitutions. The enzyme then cleaves the peptide bond between threonine and glycine to generate an acyl-enzyme intermediate. Nucleophilic attack: The free amino group of the terminal glycine in the lipid II attacks the acyl-enzyme intermediate. This forms a new amide bond between the carboxyl group of the threonine residue and the amino group of the terminal glycine, covalently linking the surface protein to lipid II. Cell wall anchoring: The lipid II-conjugated protein is then incorporated into the bacterial cell wall.

Sortase enzymes anchor proteins to the cell wall of Gram-positive bacteria. Many of these are virulence factors that must be surface-anchored for functionality, such as adhesins, or to strategically concentrate their activity where it is most beneficial to the microbe such as enzymes that defuse host immunity proteins. Recognition of proteins destined for anchoring is mediated by conserved sequence sortase called the cell wall sorting signal, LPXTG (leucine–proline–X–threonine–glycine, where X can be any amino acid) [1]. This is usually in the carboxy-terminus of the target protein (figure 1). Sortases cleave after the threonine (T) residue and catalyse a peptide bond between the cleaved fragment and the peptidoglycan in the cell wall [2]. Inhibiting sortase function can have major consequences to a pathogen, as proper localization is important for the function of virulence factors, and each pathogen can have dozens of different, individually essential, virulence factors anchored by sortase. Unlike traditional antibiotic targets, sortase inhibition is not commonly directly bactericidal, making their *in vitro* screening more of a challenge than drugs that are. However, by broadly attenuating pathogenicity, sortase inhibitors represent a paradigm shift in antimicrobial strategy—focusing on disarming the pathogen rather than killing it outright. This review discusses the complexities of treating infections caused by Gram-positive bacteria in the modern era of extensive antibiotic resistance and the current landscape of sortase inhibitors. It highlights the basic necessity for sortase-anchored proteins in key virulence processes, including adhesion, biofilm formation and immune evasion. By exploring both the promise and limitations of this approach, the review aims to provide a comprehensive understanding of sortase as a therapeutic target in the treatment of *Staphylococcus aureus* and other Gram-positive, ESKAPE or otherwise medically important pathogens.

### 1.1. Current failure of antibiotic therapy

Previously life-threatening infections by Gram-positive pathogens became treatable with the discovery of penicillin by Alexander Fleming in the 1940s [3]. However, resistance to penicillin emerged within the next 10 years with the widespread use of the antibiotic [4]. *Staphylococcus aureus* and other pathogens evolved to synthesize penicillinase, an enzyme that catalyses the β-lactam ring of penicillin, in turn, making these bacteria penicillin-resistant. The next drug introduced to treat these infections, methicillin, subsequently resulted in the evolution of methicillin-resistant *S. aureus* (MRSA) within a year of introduction [5,6]. These strains encoded a penicillin-binding protein 2a (PBP2a), which could take over the peptidoglycan synthesis when the other PBPs were inhibited by β-lactam antibiotics [7] and also had high transmission rates associated with community-acquired MRSA (CA-MRSA) or healthcare-associated MRSA (HA-MRSA) [3,8,9].

Vancomycin has been the drug of choice for years when it comes to treating multidrug-resistant Gram-positive pathogen infections, such as MRSA. Vancomycin is administered intravenously as its bioavailability is lower in oral formulations and can require hospitalization. A few other antibiotics are used to treat infections when vancomycin is no longer an option. Linezolid is approved for complicated skin and soft tissue infections and pneumonia with 100% bioavailability when taken orally, compared to vancomycin which has less than 10% [10–12]. Daptomycin is another alternative against methicillin-susceptible *S. aureus* (MSSA) and MRSA but is available through intravenous infusion only. Other antibiotic options, such as fluoroquinolones, trimethoprim/sulfamethoxazole and clindamycin, are generally recommended for superficial infections. Efficacy of newer antibiotics, such as telavancin, dalbavancin and oritavancin, has been limited to treating acute bacterial skin and skin structure infections only [13,14]. A series of factors, such as susceptibility, mode of delivery, bioavailability, side effects and severity of infection, play into the decision of using antibiotics for a patient, making it a case-by-case situation [15]. Having a spectrum of antimicrobials and widening the search beyond antibiotics is necessary to improve treatment options for every patient's unique needs.

While within the cell there is a bounty of essential processes that may be ready targets, it is often a challenge for a potential drug to cross the cell envelope. The cell wall of Gram-positive pathogens is both essential and exposed; penicillin, methicillin and vancomycin all target the proteins essential for its synthesis. Thus, the high efficacy of early antibiotics, before the evolution of resistance, may be, in part, a consequence of having a ready target. As another essential extracellular enzyme, sortase could also be such a target.

### 1.2. *Staphylococcus aureus* is a model for the essential role of sortase A

Studies, initially focused on *S. aureus*, revealed one of the earliest clues for the presence of a protein to sort or retain molecules of choice in bacteria [16]. Sec-mediated secretion provides a mechanism to transport cell surface proteins across the bacterial membrane [16]. After this, the protein can be released in a soluble form. Sortase A targets cell surface proteins containing a carboxy-terminal amino acid motif of LPXTG, where ‘X’ tolerates most substitutions for cleavage between threonine and glycine. This linkage occurs by an amide bond formed between the carboxyl group of threonine and the glycine peptide cross-bridges to anchor the protein to peptidoglycan within lipid II [16–19]. The cell surface protein gets cross-linked to the cell wall after the cleavage [20,21].

In contrast to the Gly cross-bridges of staphylococci peptidoglycan and the strict requirement for Gly in the P1′ position for its sortase A recognition, there is diversity among bacteria in this action that is linked to the cell wall composition of the species. *Enterococcus faecalis* uses L-Ala [22], *Enterococcus faecium* uses D-Asx [23] and *Streptococcus pneumoniae* uses L-Ser-L-Ala or L-Ala-L-Ala [24]. The sortase A of *Listeria monocytogenes* shares a strict preference for Gly recognition with *S. aureus*, while *S. pneumoniae*, *S. pyogenes*, *Streptococcus agalactiae*, *Streptococcus oralis*, *Streptococcus suis* and *Lactococcus lactis* and *E. faecalis* will equally tolerate other amino acids in this position, such as Ala or Ser [25,26].

While sortase A (encoded by *srtA*) acts on substrates with LPXTG motif, a second enzyme present in some species, sortase B (*srtB*), instead acts on substrates with NPQTN (asparagine–proline–glutamine–threonine–asparagine) motif in their C-terminal end [21]. Sortase A and sortase B are expressed in all clinical isolates of *S. aureus*, while the number of surface proteins they anchor to the cell wall is variable between strains [27–29]. Often 17–21 surface proteins containing LPXTG motifs are anchored to the cell wall as compared to a single iron-regulated surface protein, IsdC, containing NPQTN motif [21,30]. However, in experimental models, SrtB mutants can infect similarly to wild-type *S. aureus*, suggesting targeting sortase B is not an effective strategy to neutralize *S. aureus* [31].

While sortases are not necessary for survival of *S. aureus in vitro*, *S. aureus* lacking sortase A is significantly impaired in their ability to produce a lethal infection in several models (summarized in table 1). A much higher dose of *srtA* mutant of *S. aureus* is necessary to induce lethality, mostly likely from sepsis rather than any true virulence [20]. Mutation of individual sortase-adhered proteins can also be attenuating, suggesting sortase A decorates the surface of *S. aureus* with factors necessary to infect the host successfully. Not only infection severity but sortase also impacts whether bacteria can colonize the host. In mice, mutation in *srtA* prevents *S. aureus* from colonizing the nasopharyngeal and gastrointestinal tracts. Therefore, disabling sortase would prevent the activity of surface proteins that contribute to the success of infection without the need for targeting each of them individually.

**Table 1.** Overview of the effect of sortase gene on different animal models of infection.

species and *srt* gene mutated	animal infection model	effect	ref.
*S. aureus* Newman (wt) and *srtA* mutant SKM3 (*srtA^−^*)	tail-vein injection into (i) C57Bl/6 and (ii) Swiss-Webster mice at three different CFU to measure bacterial burden in kidney abscess	reduction (or clearance) of viable *S. aureus* from kidney abscess	[20]
*S. aureus* Newman (wt) and sortase mutant SKM3 (*srtA^−^*)	intraperitoneal injection into CD1 mice	*srtA^−^* required high CFU levels to establish lethal disease	[20]
*S. aureus* Newman (wt), *srtA* mutant SKM3 (*srtA^−^*) and SKM3 complemented with pSrtA	(i) intravenous injection into NMRI mice to study septic arthritis to examine disease progression and bacterial burden and (ii) intra-articular injection into left-knee joint to study inflammation	(i) no lethal infection with *srtA^−^* and less percentage of animals infected with *srtA^−^* develop arthritis (ii) no signs of cartilage or bone erosion or inflammation for animals injected with *srtA^−^*	[32]
*S. aureus* Newman (wt) and *ΔsrtAsrtB*	subcutaneous injection into NMRI mice	bacterial burden lower than injected inoculum in case of *ΔsrtAsrtB*	[33]
*S. aureus* Newman (wt), SKM12 *ΔsrtA*, SKM7 *ΔsrtB*, SKM14 *ΔsrtAsrtB*	intravenous injection into BALB/c mice	mortality rate lower for mice infected with *ΔsrtA* and *ΔsrtAsrtB*	[34]
*S. aureus* Newman (wt) and *ΔsrtA*, 502A (wt) and *502A ΔsrtA*	intranasal injection into ICR mice	reduced colonization of *ΔsrtA* mutants	[35]
*S. aureus* Newman (wt) and *ΔsrtA*	retro-orbital injection into BALB/c mice (10-day period)	*ΔsrtA* takes 10× more time to cause lethal infection	[36]
*S. aureus* Newman (wt), SKM12 *ΔsrtA,* SKM7 *ΔsrtB*, SKM14 *ΔsrtAsrtB*	(i) intraperitoneal injection into CD1 mice (ii) intravenous injection into NMRI mice (iii) bladder injection into C3H/HeJ mice (iv) tail-vein injection after endocarditis development in Sprague-Dawley rats	(i) mortality rate lower for mice infected with *ΔsrtA* and *ΔsrtAsrtB* (ii) lower percent of animals develop arthritis when injected with *ΔsrtA* and *ΔsrtAsrtB* (iii) reduced kidney titres for mice injected with *ΔsrtA* and *ΔsrtAsrtB* (iv) reduced cardiac titres for rats infected with *ΔsrtA* and *ΔsrtAsrtB*	[31]
*S. aureus* Newman and *ΔsrtA*	retro-orbital injection into BALB/c mice (5-day period)	reduced bacterial burden and abscess from kidney tissue in animal injected with *ΔsrtA*	[37]
*S. aureus* Newman and *ΔsrtA*	injection into right jugular vein in guinea pigs	quick recovery of animals infected with *ΔsrtA* and resolution of lower inflammatory responses and less bacterial load	[38]
*S. gordonii* SP204(1, 1) (wt) and SP-06 (*ΔsrtA*)	intranasal and oral injection into Swiss/CD1 mice	reduced colonization of *ΔsrtA*	[39]
*S. mutans* NG8 (wt) and *ΔsrtA*	inoculation into Sprague-Dawley rats to study teeth colonization	less caries when inoculated with *ΔsrtA* except for the dentinal lesion	[40]
*S. pneumoniae* D39 (wt) and *ΔsrtA*	(i) intranasal inoculation of 1:1 mix of wt and *ΔsrtA* to induce pneumonia (ii) intravenous injection (iii) nasopharyngeal colonization in MF1 mice	*srtA* gene confers advantage to *S. pneumoniae* in all disease models	[41]

### 1.3. Role of sortase in other Gram-positive pathogens

The role of sortases and their effect on pathogenesis vary among other Gram-positive bacteria. In the case of *Streptococcus* species, lack of sortase jeopardizes the distribution of surface proteins. Sortase A is one of the most abundant proteins on the surface of *S. pyogenes*, and *srtA* mutant of *S. pyogenes* has increased missorted and misfolded proteins on the cell surface [42,43]. This results in an increased sensitivity to antimicrobial peptide LL-37, showing sortase inhibitors help kill a pathogen, not only disarming them through the loss of surface-anchored virulence factors. A similar structural defect occurs in *S. agalactiae* (group B *Streptococcus*, GBS) and *E. faecalis*, wherein lack of sortase A and sortase C, respectively, abrogates proper anchorage of pili on the cell wall [44,45].

*Streptococcus agalactiae* loses its capacity to adhere to epithelial cells without expressing sortase A by 10-fold [46]. Targeted removal of srtA-anchored FbsA and FbsB surface proteins compromised binding to epithelial cells and extracellular host proteins such as fibronectin and fibrinogen [47,48]. A competitive assay reveals that *S. agalactiae* gets rapidly cleared from intestines of germ-free mice when it lacks *srtA* as compared to a strain containing *srtA* [36]. Whether sortase plays a role in colonization and persistence in humans is yet to be proven. Still sortase inhibitors could act as a first line of defence for newborns who develop severe invasive disease from *S. agalactiae*.

*Streptococcus pneumoniae* also shows a 10-fold decrease in binding to human pharyngeal cells [46,49]. In *Streptococcus gordonii*, an oral streptococcus, loss of surface proteins anchored to the cell wall by sortase may lead to an alternative compensatory mechanism to enhance biofilm formation [50,51]. Still, an *srtA* mutant shows an overall decrease in its ability to colonize the oral mucosa of mice, suggesting that sortase inhibitors could still be effective to treat infections caused by this bacterium [39]. A similar decrease in colonization occurs in the oral mucosa of mice and teeth of rats when *Streptococcus mutans* lacks sortase A [40]. This effect may also be in part owing to interspecies interactions with *Fusobacterium nucleatum*, which co-aggregates with *S. mutans* in oral biofilms in a sortase A-dependent manner [52]. Such effects are seen by streptococci pathogenic to other mammals such as *Streptococcus uberis*, a cattle pathogen, where sortase A is seen to be needed for mastitis—an economically important disease affecting the mammary glands of cows in dairy farms [53,54].

Still less is known in other species. Another Gram-positive pathogen, *Bacillus anthracis*, also harbours sortase A-anchored surface proteins that are potentially involved in anthrax [55]. However, whether those proteins have a role in disease is yet to be understood [56]. Sortase also plays a role in *L. monocytogenes*, wherein an *srtA* mutant lacks surface virulence factors and is attenuated in a mouse model of infection that is lethal by a wild-type strain [57]. Sortase inhibitors may not be a universal antimicrobial to treat all Gram-positive pathogens to the same level. However, they can reduce disease progression to a varying extent in these pathogens as they affect adhesion, colonization and virulence.

### 1.4. Sortase-anchored adhesins

**Figure 2.**
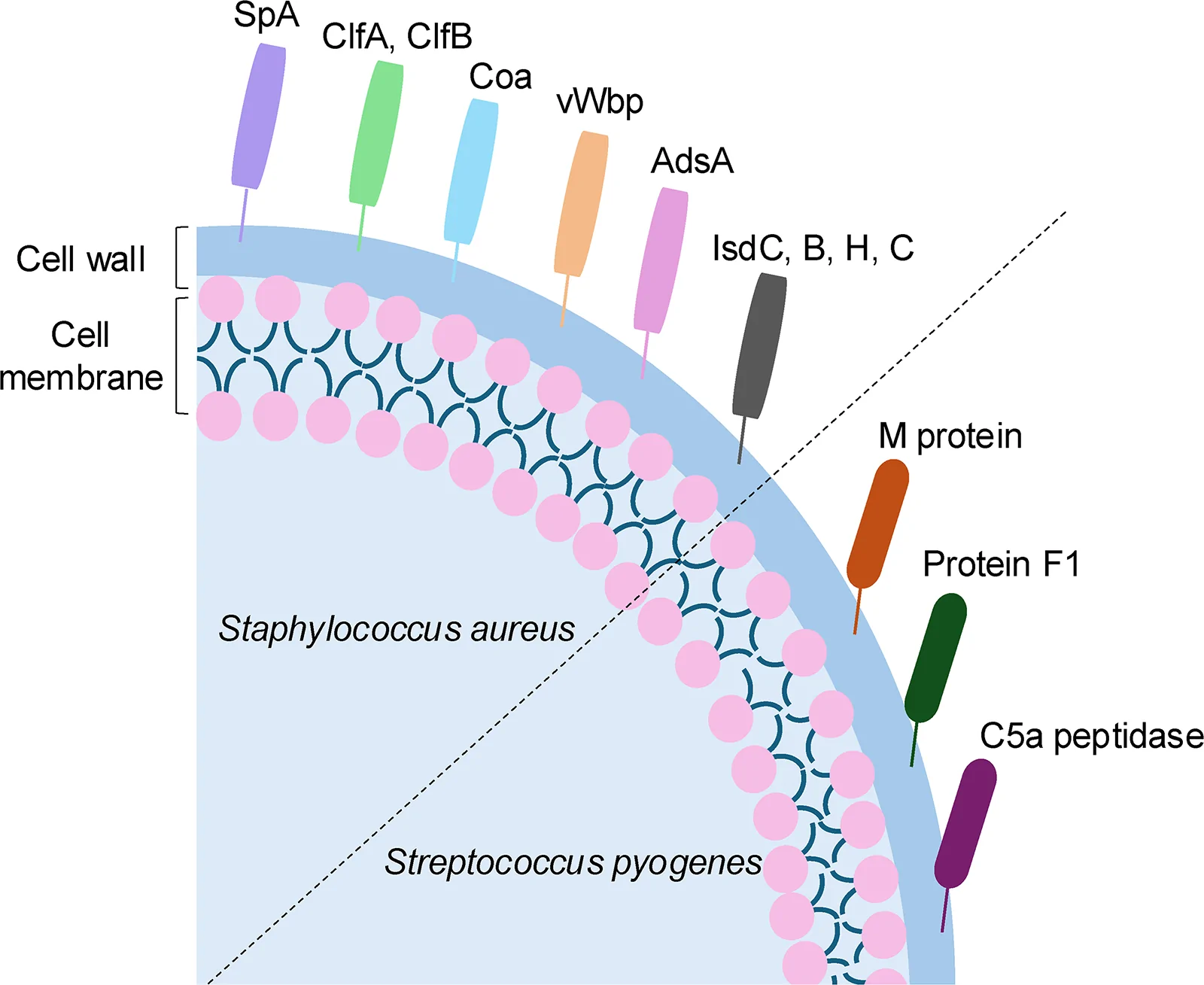
Major sortase A-anchored MSCRAMMs of *Staphylococcus aureus* and *Streptococcus pyogenes*. The sortase A-anchored proteins include staphylococcal protein A (SpA), clumping factor (ClfA and ClfB), coagulase (Coa), von Willebrand factor-binding protein (vWbp), adenosine synthase A (AdsA), iron-regulated surface determinant (IsdA, IsdB, IsdC and IsdH) in *Staphylococcus aureus* and M protein, protein F1, C5a peptidase in *Streptococcus pyogenes*.

One broad grouping of sortase substrates are MSCRAMMs (microbial surface components recognizing adhesive matrix molecules), which are anchored to the cell wall to promote adhesion during infection through a variety of strategies [58,59]. These are a major group of sortase-anchored adhesins essential to host–pathogen interaction and immune evasion. Extensive literature about MSCRAMMs comes from studies in *S. aureus*, though examples are known for *Staphylococcus epidermidis*, *Streptococcus pyogenes* and other streptococci and staphylococci (table 2 and figure 2). Many of these species encode additional adhesins, such as pilin proteins [99], that require sortase B or C, which are more targeted to specific substrates, and as noted earlier, would give less broad inhibition of virulence activities relative to sortase A if targeted.

**Table 2.** Overview of major sortase-anchored adhesins in *Staphylococcus aureus* and *S. pyogenes* acted upon by sortase A.

protein name	ligand	ref.
***Staphylococcus aureus***		
staphylococcal protein A (SpA)	Fc region of IgG and F-ab domain of V_H_3-type B-cell receptor	[60,61]
clumping factor (ClfA and ClfB)	ClfA binds to fibrinogen, fibrin and Annexin A2 ClfB binds to fibrinogen, cytokeratin 10 and loricrin	[62–67]
coagulase (Coa)	fibrinogen and prothrombin	[68,69]
von Willebrand factor-binding protein (vWbp)	fibronectin and von Willebrand factor	[70–72]
adenosine synthase A (AdsA)	adenosine binds to A1, A2A, A2B and A3 receptors	[73–75]
iron-regulated surface determinant (IsdA, IsdB, IsdC and IsdH)	IsdB binds free haem, haemoglobin–haptoglobin and vitronectin IsdH binds free haem and haemoglobin–haptoglobin IsdA and IsdC shuttle haem via ABC transporter IsdE	[76–78]
fibronectin-binding protein (FnbpA and FnbpB)	fibronectin, fibrinogen, elastin, plasminogen, loricrin	[79–83]
***Streptococcus pyogenes***		
M protein	fibrinogen, factor H, C4b-binding protein (C4BP), immunoglobulins, plasminogen, LL-37, histones within neutrophil extracellular traps	[84–92]
protein F1	fibronectin	[93–96]
C5a peptidase	human complement protein C5a	[97,98]

Staphylococcal protein A (SpA), anchored by sortase in *S. aureus*, gives the capacity to deflect both innate and adaptive immune responses. SpA binds to both Fcγ domain of an immunoglobulin and F-ab domain of V_H_3-type B-cell receptor [60,61]. The interaction between SpA and Fcγ causes antibodies to orient in reverse on the bacterial surface relative to the requirement for normal opsonization [100]. As a result, *S. aureus* evades phagocytosis from innate immune cells such as macrophages and neutrophils. On the other hand, SpA's binding to F-ab region triggers B-cell superantigen activity [101–103]. However, this interaction enhances *S. aureus* virulence and is not protective to host [104,105]. On disrupting SpA's binding to Ig in mice, immune cells phagocytosed *S. aureus* and elicited B-cell responses helpful in protecting mice from the infection [106]. Thus, one effect of sortase inhibitors would be to limit the ability of *S. aureus* to evade antibodies and opsonophagocytosis, improving their clearance from host. A plant product, cyanidin chloride, was able to disrupt SpA's binding to Ig by inhibiting sortase A activity and improved the survival rate of mice with MRSA-induced pneumonia [107].

Additionally, *S. aureus* manipulates coagulation system and directly activates abnormal clot formation using three sortase-anchored proteins: coagulase (Coa), von Willebrand factor-binding protein (vWbp) and clumping factor A (ClfA) [36,70,108]. Coa and vWbp activate host prothrombin to thrombin. Thrombin, then, converts fibrinogen to fibrin to coagulate plasma. ClfA adheres to fibrinogen and fibrin to promote bacterial agglutination or clumping. Conventional coagulation inhibitors fail to reverse these pathological complications [109]. Sortase inhibitors will be able to prevent this survival strategy by preventing Coa, vWbp and ClfA from creating the clumping sites. The lack of these surface proteins in a mouse sepsis model failed to infiltrate agglutination in heart tissues, suggesting their importance [36]. Another clumping factor, ClfB, adheres *S. aureus* to loricrin and cytokeratin 10 present on nasal cells [62]. Sortase inhibition will inhibit pathologic coagulation and colonization and will improve perfusion of conventional antibiotics and immune antimicrobials to the infection site [110].

*Staphylococcus aureus* uses AdsA, another sortase-anchored virulence factor, to counteract the primary defence mechanisms of neutrophils. First, AdsA elevates adenosine levels to promote survival of *S. aureus* during infections by dephosphorylating ATP, ADP and AMP [73]. Adenosine serves as an important molecule to suppress immune responses by counteracting excessive host inflammatory responses [111–118]. An excess of adenosine produced by AdsA during *S. aureus* infections hijacks the host's immunosuppressive pathway and dampens neutrophil and T-cell function to its own benefit [114]. AdsA also degrades extracellular DNA from the 5 and 3 monophosphate nucleotides into deoxyadenosine [73,119]. The deoxyadenosine enters macrophages via a transporter protein and converts to dATP and triggers apoptosis in them [74,119]. Sortase inhibition can, therefore, restore immune function against *S. aureus* and revert the damaging effects of AdsA.

Host factors limit access to metals such as iron, zinc and copper by sequestering them away from invading microbes. *Staphylococcus aureus* procures iron from haem primarily using Isd proteins [120–122]. Sortase A anchors three Isd proteins, including IsdA, IsdB and IsdH, and sortase B anchors IsdC on the peptidoglycan layer. IsdB and IsdH bind free haem and haemoglobin–haptoglobin, whereas IsdA and IsdC shuttle haem via ABC transporter IsdE [76]. Lack of *isdB* impairs staphylococcal growth by preventing iron uptake from haemoglobin [121]. Also, *srtA* mutant of *S. aureus* loses its ability to control iron levels to prevent toxicity [121,123]. IsdB can also bind to vitronectin and promote binding of *S. aureus* to epithelial and endothelial cells and act in favour of *S. aureus* in more than one way [77]. To that end, a naturally occurring compound, baicalin, has already been shown to disrupt binding to human alveolar epithelial cells by inhibiting SrtB. Additionally, sortase inhibitors will prevent *S. aureus* from attaining iron homeostasis and, hence, limit the bioavailability of ferrous form of iron for successful nutrient acquisition [124].

Similar to *S. aureus*, *S. pyogenes* also harbours a number of sortase-anchored surface proteins essential to colonize host tissue, establish infection and counteract immune killing, including M protein, protein F1 and C5a peptidase [125]. M protein, a major surface protein of *S. pyogenes*, adheres to extracellular matrix proteins such as fibrinogen, fibronectin, laminin, and collagen, immunoglobulins, cathelicidin (LL-37) and histones to mask itself from immune recognition [84–86,126]. M protein forms complexes with complement factors and immunoglobulins that prevent phagocytosis of *S. pyogenes* [87,88,127–129]. M protein also elicits cross-reactive antibodies to myosin, tropomyosin and keratin, leading to molecular mimicry and immune evasion [130]. An M mutant of *S. pyogenes* is attenuated in human blood and mouse infection models [85,86,131]. Sortase inhibitors can neutralize M protein's multi-functionality that contributes to the versatility of *S. pyogenes* as a pathogen. M-like proteins, such as protein F1 (PrtF1/SfbI), also function similarly to M protein and improve adherence to epithelial cells [93–95]. C5a peptidase is also an M-like protein that inactivates completement protein, C5a, a chemoattractant for neutrophils [97]. Thus, a major effect of sortase inhibitors in improving host survival can come from impairing the functions of M protein and M-like proteins, but numerous other virulence factors may be impacted.

All Gram-positive pathogens mediate binding to cells and initiate infection using surface proteins. The aforementioned examples are among the many homologous proteins that sortase inhibitors can hinder and provide therapeutic benefit against.

## 2. Biofilms

Bacteria have the unique ability to adhere to each other by synthesizing extracellular polymeric substances and proteins. Both staphylococcal and streptococcal biofilms complicate infections as they prevent antibiotic penetration, making treatment challenging [110,132]. Bacteria create dormant populations of biofilms in medically implanted devices and can lead to serious infections impervious to any treatment strategies [133,134]. *Listeria monocytogenes* can also take advantage of biofilm formation to survive long term on food packaging and cause illnesses later [135]. Sortase A and other sortase family proteins play an indirect role in biofilm formation as they help to create the building blocks of biofilms and can be induced in the biofilm state [136].

Host proteins such as fibronectin and fibrinogen coat plastic- and metal-based medical devices, in turn, initiating biofilm formation by sortase-anchored proteins [137,138]. Proteins such as fibronectin-binding proteins (FnbpA/B), SasG in *S. aureus* and homologous proteins such as SdrG/Fbe and RP62a in *S. epidermidis* promote biofilm formation to advanced stages [137,139–141]. In *S. mutans*, biofilms are key to forming dental caries [142]. Targeting sortase could be a successful therapeutic strategy as inactivation of sortase-anchored proteins resulted in a one-fourth reduction in total biofilm biomass in *S. mutans* [142,143]. An *srtA* mutant of *S. mutans* no longer binds to saliva, suggesting a clear role of sortase in colonization and biofilm formation [143]. Natural products with antibiofilm activity are already in pipeline against *S. mutans* and *S. aureus* [144–148]. Genistin, a natural plant product, has also been investigated to inhibit biofilm in *L. monocytogenes* [149].

## 3. Advantages and disadvantages of sortase inhibition

The premise of antibiotic therapy continues with the need to discover new bacterial targets. The development of sortase inhibitors takes an alternative route wherein the focus becomes the necessary virulence factors for disease progression. Strategies to target key virulence factors may not directly kill *S. aureus* but can undo the damage inflicted on the immune system. This, then, can allow the clearance of *S. aureus* by host innate and adaptive immune responses. The high conservation of sortase enzymes and their targets, and surface localization meaning cell permeability is a lesser concern, makes them excellent drug targets. Furthermore, developing resistance against sortase inhibitors would require *S. aureus* and other Gram-positive pathogens to develop a completely new strategy to identify the conserved motif on the surface proteins—mutating surface proteins to have a new cell wall sorting signal or repurposing an alternate sortase.

Thus, inhibitors targeting the cysteine or other catalytic residues of sortases, or those coordinating interaction with the substrate leucine, proline and threonine residues, may have broader activity than those impacting cell wall interactions. However, narrower species specificities could be desirable to avoid impacts on non-pathogen resident species of the microbiota.

As discussed earlier, one benefit of sortase inhibition is the inactivation of numerous surface-anchored proteins important for virulence. However, an important consideration in some circumstances may be whether this leads to secretion of the impacted proteins from the bacterium. In the case of protein A of *S. aureus*, release by murein hydrolases allows secretion and binding of immunoglobulins via their Fc domain and act as a B-cell superantigen [150]. M protein can also be released from cells and activate the NLRP3 inflammasome, resist killing by neutrophil extracellular traps and LL-37 and overstimulate neutrophils to release heparin-binding protein to cause severe tissue damage [85,86,151–156]. Thus, a treatment that leads to a rapid and extensive release of molecules has the potential to promote inflammation, oedema and necrosis [157]. In the context of bacteraemia or another sterile-site infection, this could lead to sepsis, regardless of bacterial killing. For example, β-lactam antibiotic treatment can cause bacteria to release a bolus of peptidoglycan fragments, which are highly proinflammatory [158]. In contrast, protein synthesis inhibitors often reduce inflammation by blocking the production of toxins otherwise induced by the stress responses during treatment [110].

## 4. Attempts at sortase inhibition

Early attempts focused on repurposing existing drugs to inhibit sortases, but those experiments revealed more about its enzyme activity and less about its viability as anti-infectives. For example, both vancomycin and moenomycin delayed sorting of proteins, whereas penicillin did not affect it. These drugs showed that sortase depends more on peptidoglycan precursors than on the fully assembled cell wall of *S. aureus.* Chemically synthesized inhibitors—methanethiosulfonates and *p*-hydroxymercuribenzoic acid—showed sortase needed the sulfhydryl group of lipid II substrate formed from the transpeptidation [159]. These experiments provided indirect proof on how sortase inhibitors could function as anti-infectives. Several recent reviews can provide additional detail on the chemical strategies to develop sortase inhibitors [148,160–164]. Here, we describe a few of the recent and historical studies important for understanding some of the breadth of this field.

### 4.1. Types of sortase A inhibitors

#### 4.1.1. Synthetic molecules

Scott *et al.* synthesized chemical inhibitors that serve as analogues to sortase A specifically [165]. These drugs included peptidyl-diazomethane and peptidyl-chloromethane, active against SrtA. Another group of inhibitors included vinyl sulfone substrates that mimicked the LPXTG substrate to block SrtA activity [166]. These act as electrophilic inhibitors and react with the cysteine residue of SrtA to form a permanent covalent bond [167,168]. However, these early molecules were specifically targeting SrtA; rather the focus was to use these compounds to better understand the function of SrtA. Hence, they were pursued rigorously for *in vitro* and *in vivo* studies.

#### 4.1.2. Natural products

Naturally occurring compounds, such as those from plants, have been shown to be a fruitful source to isolate sortase inhibitors. β-Sitosterol-3-*O*-glucopyranoside from *Fritillaria verticillata* as well as berberine chloride from *Coptis chinensis* had inhibitory effect on *S. aureus*, *Bacillus subtilis* and *Micrococcus luteu*s [169,170]. The same research group also pivoted their knowledge in identifying natural products against both SrtA and SrtB in a marine product library to find psammaplin A1 from *Aplysinella rhax* [171]. Curcumin and derivatives from *Curcuma longa* inhibited binding of *S. aureus* to fibronectin—proving an early clue that virulence factors are indeed targeted by sortase inhibitors [172]. Other natural compounds include polyphenols such as curcumin, quercetin, dryocrassin, wogonin, daphnoretin and luteolin among others [173,174].

### 4.2. Methods for finding sortase A inhibitors

High-throughput screening (HTS) provides automation for screening a compendium of molecules at once. It accelerates discovery of drug candidates and includes a number of strategies such as fluorescence-based methods and cell adhesion and clumping assays. Multiple HTSs have been performed on small molecule libraries for the discovery of sortase inhibitors.

Oh *et al.* found a diarylacrylonitrile through a random screen of a small molecule library with 1000 compounds using a recombinant SrtA [175]. This molecule was tested along with β-sitosterol-3-*O*-glucopyranoside, berberine chloride and psammaplin A1 for comparable defects of *S. aureus* binding to fibronectin [171]. The same molecule was later shown to be protective in reducing kidney and joint infections of *S. aureus* by 90% in mice [34]. Another HTS revealed around 407 compounds that could target different sortase family between species [176]. Over the years, β-amino(ethyl) ketones (AAEK) and pyridazinone-based molecules are another unique class of molecules that were discovered to disrupt the catalytic activity of sortases [177–179]. Virtual screening, almost complementary to HTS, depends on the screening library pre-existing in databases. In a study performed in 2014, Zhang *et al.* discovered a class of 3,6-disubstituted triazolothiadiazole compounds to have potent inhibitor activity against sortase [180]. One of the compounds prevented the lethal outcome of bacteraemia in BALB/c mice [180]. Another recent study used a hybrid virtual screening method to identify five compounds. One of their compounds, 2-(2-amino-3-chlorobenzoylamino)benzoic acid, showed antibacterial effects and inhibited biofilm formation in *S. aureus* isolates from hospitals [181].

*Actinomyces oris* is one of the only known bacteria where sortase appears to be essential, which enabled the development of a cell-based assay to screen for sortase inhibitors. Reduced expression of SrtA enzyme leads to the accumulation of a glycosylated surface protein (GspA) in the membrane and toxicity. Based on this process, Gosschalk *et al.* screened more than 200 000 molecules that impair *A. oris* [182]. Then, selected molecules were counter-screened with Δ*srtA*/Δ*gspA A. oris* followed by validations using biochemical and cellular approaches. This approach led to the discovery of three molecules of interest that were found to inhibit sortases from both *S. aureus* and *A. oris*.

## 5. Conclusion

When it comes to the fight against antibiotic resistance, we are nowhere close to an end. Antibiotic discovery efforts have been primarily driven by the approach of inhibiting bacterial growth by targeting conserved, essential mechanisms, typically in artificially designed mediums not mimicking natural host environment. Such a strategy prioritizes many poor targets within the cell that are difficult for drugs to access and ignores many excellent targets, such as sortase A, which is not required for growth in rich media but essential for a pathogen during infection. Furthermore, sortase inhibition carries the advantage of not focusing on single virulence factors, which are variable between species and strains, but by inactivating a broad repertoire of essential factors for many or most Gram-positive pathogens. Therefore, sortase inhibitors bridge the gap between the bacterium and host by inhibiting virulence determinants central to the infectious process. Investigation of surface proteins at the interface of host and bacterium can open a wave of possibilities in the area of anti-infective therapies.

The first step to developing sortase inhibitors starts with the need to learn the intricacies of the decoration of surface proteins by sortase. There are numerous outstanding questions that remain to be understood. Are the same surface proteins present at every step of infection by *S. aureus* and other pathogens? Does the repertoire of surface proteins expressed differ based on the site and severity of infection? Can sortase inhibitors prevent the expression of surface proteins at all stages of infection and decrease bacterial survival indirectly? Can these inhibitors be useful as a monotherapy, or are they useful in combination with antibiotics? What resistance mechanisms will evolve and how quickly? Will these drugs have negative impacts on the microbiota? Finally, it will be necessary to learn whether sortase inhibitors can alleviate the damage inflicted by surface proteins and allow the immune system to function effectively for better therapeutic outcomes.

## Data Availability

This article has no additional data.
